# Maternal childhood emotional abuse increases cardiovascular responses to children’s emotional facial expressions

**DOI:** 10.1371/journal.pone.0302782

**Published:** 2024-05-07

**Authors:** Rachel Pétrin, Annie Bérubé, Émilie St-Pierre, Caroline Blais

**Affiliations:** Department of Psychoeducation and Psychology, Université du Québec en Outaouais, Gatineau, Québec, Canada; Harvard Medical School, UNITED STATES

## Abstract

Parents with a history of childhood maltreatment may be more likely to respond inadequately to their child’s emotional cues, such as crying or screaming, due to previous exposure to prolonged stress. While studies have investigated parents’ physiological reactions to their children’s vocal expressions of emotions, less attention has been given to their responses when perceiving children’s facial expressions of emotions. The present study aimed to determine if viewing facial expressions of emotions in children induces cardiovascular changes in mothers (hypo- or hyper-arousal) and whether these differ as a function of childhood maltreatment. A total of 104 mothers took part in this study. Their experiences of childhood maltreatment were measured using the Childhood Trauma Questionnaire (CTQ). Participants’ electrocardiogram signals were recorded during a task in which they viewed a landscape video (baseline) and images of children’s faces expressing different intensities of emotion. Heart rate variability (HRV) was extracted from the recordings as an indicator of parasympathetic reactivity. Participants presented two profiles: one group of mothers had a decreased HRV when presented with images of children’s facial expressions of emotions, while the other group’s HRV increased. However, HRV change was not significantly different between the two groups. The interaction between HRV groups and the severity of maltreatment experienced was marginal. Results suggested that experiences of childhood emotional abuse were more common in mothers whose HRV increased during the task. Therefore, more severe childhood experiences of emotional abuse could be associated with mothers’ cardiovascular hyperreactivity. Maladaptive cardiovascular responses could have a ripple effect, influencing how mothers react to their children’s facial expressions of emotions. That reaction could affect the quality of their interaction with their child. Providing interventions that help parents regulate their physiological and behavioral responses to stress might be helpful, especially if they have experienced childhood maltreatment.

## Introduction

Dysregulation of human physiological responses is one of the many consequences of childhood maltreatment [1]. Indeed, prolonged exposure to stressful events during childhood has been shown to alter the normal functioning of neurobiological systems responsible for brain maturation, cognitive development, and emotional and behavioral regulation [2]. Accordingly, changes in the cardiovascular system can be observed following the experience of childhood maltreatment. However, the literature on this topic needs to be clarified, as repeated exposure to stress in the first years of life can result in under-arousal in some individuals and over-arousal in others. Nonetheless, when one becomes a parent, the disruption of physiological reactivity may represent a risk factor for perpetuating childhood maltreatment, mainly because of its suspected association with maladaptive parenting [3]. Therefore, understanding how different situations and contexts affect parental autonomic reactivity is paramount.

The autonomic nervous system (ANS) regulates physiological processes such as heart and respiratory rates. Two subsystems comprise the ANS: the sympathetic nervous system (SNS) and the parasympathetic nervous system (PNS). The SNS mobilizes the individual’s resources to prepare the body to fight or flee during stress or emergencies. In contrast, the PNS controls homeostasis and the resting and digestive response of the body, reducing physiological arousal. A typical stress response involves the activation of the SNS and the inhibition of the PNS (i.e., vagal withdrawal), allowing the individual to manage the threat and return to normal functioning once it has ended [3]. Maltreated children are often exposed to constant stress, which can lead to ongoing activation of the SNS and become a chronic issue over time due to increased allostatic load, also described as a build-up of chronic stress [4]. As a result, two types of dysfunctional physiological responses may emerge in response to a stressful event, either hypo- or hyper-arousal. A reduced physiological activation characterizes a hypoarousal, whereas a hyperarousal reflects an intense physiological activity and a slower recovery.

The respective activity of the SNS and the PNS can be measured using cardiac indicators. A direct measure of SNS activation is the pre-ejection period (PEP), defined as the period between the time when the ventricles of the heart contract and the ejection of blood into the aorta, reflecting cardiac contractility [5]. Heart rate (HR), skin conductance, and systolic or diastolic blood pressure are indirect measures of changes in SNS activity. Decreased PEP, increased heart rate, and increased blood pressure or skin conductance levels indicate activation of the SNS. Furthermore, heart rate variability (HRV) metrics assess PNS activity and are divided into three main domains: time, frequency, and geometric [6]. While heart rate (HR) is defined by the number of heartbeats per minute, HRV represents the fluctuation in the time intervals between adjacent heartbeats [7]. The activation of the SNS results in an elevated heart rate, which reduces the beat-to-beat interval and leads to decreased HRV. Conversely, the dominance of the PNS leads to reduced heart rate and increased HRV. The root mean square of successive differences (RMSSD) is the primary time-domain measure to estimate the parasympathetically mediated changes in HRV [8]. RMSSD reflects short-term beat-to-beat variance in HR and thus reflects PNS activity [9]. A higher RMSSD corresponds to an increased HRV, suggesting PNS activation. Another metric for HRV is Respiratory Sinus Arrhythmia (RSA).

The physiological reactivity of adults with a history of childhood maltreatment has been compared to that of typical adults when exposed to psychosocial stressors (e.g., public speaking). Results indicate chronic hyperreactivity in women with a history of maltreatment; that is, they would display more pronounced physiological stress responses than controls [10–12]. However, some studies have also shown that individuals with a history of maltreatment tend to have blunted cardiovascular reactivity to stress tasks [13, 14]. For example, Ginty et al. [15] observed that individuals with a history of abuse, mainly driven by sexual abuse, exhibited diminished cardiovascular reactivity (hyporeactivity) upon exposure to acute psychological stress. Additionally, adverse childhood experiences (ACEs) have been linked to reduced cortisol and heart rate responses to stress, with the overall duration of ACEs being associated with a reduction in cortisol response [16].

Moreover, Buisman et al. [17] highlighted differences in parents’ autonomic responses based on their childhood maltreatment experiences during a parent-offspring conflict interaction task. Higher levels of childhood neglect among parents resulted in a decreased RSA and a shorter PEP, reflecting autonomic hyperreactivity and lower basal RSA, denoting chronic cardiovascular hyperreactivity. Parents who experienced childhood abuse did not show abnormal stress responses during the task.

Very few studies have examined parents’ autonomic reactivity and regulation in response to children’s emotional stimuli [18–20]. For example, Casanova et al. [18] observed that the cardiovascular responses of mothers with a history of childhood abuse did not differ significantly from those without a history of childhood abuse when viewing videotapes of smiling or crying infants. However, an inverse type of response was found in the skin conductance of the two groups. Unlike mothers without a history of childhood abuse, the skin conduction of mothers with a history of childhood abuse increased (indicating increased SNS activity) when viewing a video of a smiling infant but not of a crying infant. These results suggest that children’s positive emotional responses may hold stressful properties for mothers who experienced childhood abuse, and these mothers may be less sensitive to children’s negative emotions. Furthermore, it has been shown that PEP recovery patterns differ according to childhood maltreatment experiences in non-maltreating mothers [20]. An increasing trend in PEP values (i.e., values during a 4-min neutral image baseline after exposure to the cry paradigm) was found in mothers with more maltreatment experiences compared to those with few or no maltreatment experiences whose PEP values tended to decrease (reflecting increased SNS activity). On the other hand, Buisman et al. [19] did not uncover a difference in autonomic reactivity in parents with a history of childhood neglect when exposed to infant emotional signals. Instead, parents with a history of neglect exhibited a higher HR and shorter PEP throughout the infant vocalization paradigm, which may represent chronic cardiovascular hyperreactivity. In conclusion, studies measuring parents’ autonomic responses to psychosocial stressors and infant emotional signals have shown that different types of childhood maltreatment (i.e., abuse or neglect) affect parental autonomic responses in varying ways.

Most of the work on autonomic reactivity in parents with a history of childhood maltreatment has been conducted using the auditory perception of the child’s distress (hearing infant cries) rather than the visual perception of children’s faces. As children get older, they become more proficient at handling different emotions. By age two, they can feel and express various emotions comparable to adults and have better control over them [21]. Thus, children can increase the delay between expressing an emotion and its externalization through crying and bursts of laughter or anger, for example. During this short period, parents have the opportunity to adjust their behavior in order to respond appropriately to the child. Their success in doing so relies on their ability to recognize their child’s emotions, their interpretation of the situation as urgent, and their physiological response to the potential stress.

This paper examines whether mothers’ childhood maltreatment experiences contribute to their physiological responses when perceiving facial expressions of emotions in children. More specifically, we seek to determine if viewing children’s facial expressions of emotions compared to a landscape video induces autonomic hypo- or hyper-reactivity in mothers with a history of maltreatment and if the response differs according to the forms of childhood maltreatment experienced. Understanding how a history of childhood maltreatment affects parents’ physiological responses to children’s facial expressions of emotions is important in explaining why parenting is often more challenging for those parents than for others. This knowledge can shed light on the mechanisms contributing to the intergenerational maltreatment cycle [22, 23].

## Materials and methods

The present study was approved by the Ethical Committee of the University of Quebec in Outaouais (UQO CER #2518 and 2020–698). All tasks were carried out following the university’s guidelines and regulations. Written informed consent was obtained from all subjects before their involvement in the study, which was conducted between April 2017 and September 2023.

### Participants

Participants of the current study were part of a more extensive study on the role of emotion perception in the intergenerational transmission of maltreatment [24, 25]. The inclusion criterion for mothers and children was oral and written proficiency in French, and the exclusion criterion was a diagnosis of developmental delay. The total sample included 104 mothers. Participants with heart problems or taking heart or blood pressure medication were excluded from the present study because of its focus on cardiovascular responses (n = 10). Due to technical difficulties with the electrocardiogram (ECG), HRV data were missing for 14 participants. Likewise, information on childhood maltreatment was unavailable for one of the mothers. The final sample included 79 mothers (*M* = 33.99 years, *SD* = 5.46) and their 2- to 5-year-old child (*M* = 49.32 months, *SD* = 14.50). Recruitment was carried out in community organizations that provide services to vulnerable families, social media (Facebook and Instagram), and posters in the community and on the university’s walls. Most participants were Caucasian (83.8%) and had completed post-secondary education (67.1%), although some (15.2%) did not have a high school diploma. Half of the mothers had a full-time job (48.8%), and almost half reported having an annual income of less than 24 000$ CAD (43.2%). As for the children, there were nearly equal numbers of girls (53.8%) and boys (45.0%). Most mothers (65.8%) reported experiences of childhood maltreatment (scoring at least low maltreatment in one of the five forms of maltreatment).

### Procedure

First, participants’ consent was obtained for an experiment lasting approximately two and a half hours; tasks related to the present study took about an hour to complete. Following their arrival at the university or the community centers they attended, dyads were separated into two rooms. Before undertaking the tasks, all mothers completed a sociodemographic information form and a questionnaire about their consumption habits. The ECG electrodes were then placed on the parents, and they watched a five-minute landscape video on the computer screen. Next, mothers had to perform a computerized task in which they were exposed to a bank of images showing children’s faces expressing different intensities of emotion. Throughout this task, mothers’ heart rate was recorded continuously to obtain a measure of the physiological activation induced by each of the images presented. After the recording, they were invited to complete a questionnaire about their childhood maltreatment experiences. Participants received $40 CAN for their participation in this research project.

### Measures

#### Demographic information

Mothers completed a self-reported questionnaire that collected general information such as the gender and age of the child, the mother’s age, ethnicity, education, family status, participants’ income, and other relevant information. This questionnaire version holds 42 questions and is from the *Place aux Parents* tool [26]. Mother’s last completed level of education was rated on a five-point Likert scale (1 = *Primary*, 2 = *Secondary*, 3 = *Diploma of Vocational Studies (DVS)*, 4 = Collegial, 5 = *University*).

#### Consumption habits

Participating mothers were asked to complete a short, self-reported questionnaire about their smoking habits. The questionnaire asked whether the participant currently smoked cigarettes (1 = *Every day*, 2 = *Occasionally*, 3 = *Not at all*) and, if so, when they last smoked. Participants were also asked when they last engaged in physical activity, if at all. Finally, the questionnaire included questions on a heart problem diagnosis in the past and the use of heart or blood pressure medication. This information was collected to control any possible influence on the ECG data. This 22-item questionnaire contains questions from the Quebec Longitudinal Study of Child Development [27].

#### Childhood maltreatment

The French version of the Childhood Trauma Questionnaire (CTQ) [28] was used to verify the presence of childhood maltreatment experiences in mothers. This self-reported questionnaire comprises 28 questions with a Likert-type scale (1 = *Never true* to 5 = *Very often true*). The CTQ provides a score of childhood maltreatment experiences by evaluating the five forms: emotional neglect, physical neglect, emotional abuse, physical abuse and sexual abuse. The CTQ has been validated with a large population of respondents and has good test-retest reliability (r between 0.76 and 0.96). In addition, the short version of the CTQ (28 items) has been validated by more than one study [28, 29] and is usable with a French-speaking population. Each type of maltreatment corresponds to five items, and there are three validation items. To determine the reported severity of maltreatment experiences, the scores of the five items for each type of maltreatment are summed up, resulting in a total score ranging from 5 to 25. The internal consistency for our sample is α = .88.

#### Autonomic responses to facial expressions of emotions in children

During the presentation of a landscape video (baseline) and facial expressions of emotions in children, ECG signals were recorded using the Acqknowledge system from Biopac (version 3.8). The ECG signal was recorded continuously using three disposable pre-gelled electrodes Ag/AgCl (Biopac, Canada). The first electrode (RA) was placed at the base of the right shoulder, about two centimeters below the clavicle. The second and third electrodes were placed on the left (LL) and right side (RL), respectively, halfway between the last rib and the iliac crest. The physiological data was integrated by a model MP36r device and was analyzed with the Acqknowledge software. The standard error of measurement is less than two beats/minute. Each ECG recording was visually inspected, and trained research assistants manually corrected abnormal beats and artifacts when necessary. The analysis tool of Acqknowledge automatically computed RMSSD values extracted by fixed time intervals (Multi-epoch HRV–Statistical) and mean heart rate for both conditions.

#### Perception of children’s facial expressions of emotions

The stimuli were presented on a computer screen, 40 centimeters (cm) from the participants’ faces. Mothers were exposed to numerous photographs of two Caucasian children, a boy aged 4.6 years and a girl aged 5.3 years. The faces were taken from The Child Affective Facial Expression (CAFE) database [30] and were approximately 13 cm by 13 cm in size. These images were presented in different levels of gray on a neutral gray background as they help to process facial expressions rather than low-level proprieties. The six emotional expressions under study, anger, disgust, fear, joy, sadness, and surprise, were presented to the mothers in a randomized order.

### Statistical analyses

Descriptive statistics and bivariate correlations were computed for all study variables with SPSS (version 29). The delta HRV was calculated by subtracting mothers’ HRV when viewing facial expressions of emotions in children from mothers’ HRV when viewing a landscape video (baseline). Based on the delta scores, participants presented two different profiles that were used to form two groups. Mothers with a diminished HRV during the presentation of emotions compared to landscapes (*n* = 39) were assigned to the reduced HRV group (Group 1). Group 2 included mothers with an increased HRV during the emotional component of the task (*n* = 40). A mixed model analysis of variance (ANOVA) was performed to verify the impact of belonging to one of these two HRV groups according to the types of childhood maltreatment experienced by mothers. This model was selected because it accounts for the shared variance among the different forms of maltreatment.

## Results

### Descriptive statistics

Descriptive statistics and bivariate correlations were calculated using the SPSS software (version 29) for all study variables and are presented in Table 1. The two groups did not differ significantly in terms of mother’s age [*t* (77) = .10, *p* = .92], child’s age [*t* (77) = .03, *p* = .98] and mother’s education [*t* (74.52) = 1.56, *p* = .12]. The means of other variables, such as physical exercise, smoking, and coffee and alcohol consumption, were alike for both groups (*p* > .05).

**Table 1:** Descriptive statistics and intercorrelations (n = 79).

Variables	1	2	3	4	5	6	7	8	9	10	11	12	M (*SD*)	Skewness (*SE* = .27)	Kurtosis (*SE* = .54)
1. Mothers’ age	-												33.99 (5.46)	.04	-.36
2. Mothers’ education	.32**	-											3.62 (1.58)	-.59	-1.34
3. Physical exercise	-.14	-.11	-										1.58 (.50)	-.34	-1.93
4. Caffeine use	-.08	-.19	.02	-									1.47 (.50)	.13	-2.04
5. Alcohol use	-.07	-.22	.07	.16	-								1.71 (.46)	-.92	-1.19
6. Smoking	.15	.44**	-.08	.00	-.10	-							1.81 (.40)	-1.61	.61
7. Physical abuse	-.03	-.38**	.05	.01	.15	-.16	-						6.89 (4.08)	2.58	6.55
8. Emotional abuse	.06	-.31**	.06	-.07	.10	-.15	.74**	-					8.97 (5.41)	1.73	2.18
9. Sexual abuse	.01	-.26*	-.03	.13	.13	-.13	.58**	.38**	-				6.76 (3.79)	2.98	9.76
10. Emotional neglect	.06	-.29**	-.06	-.03	.17	-.01	.71**	.78**	.46**	-			10.20 (5.04)	1.11	.43
11. Physical neglect	.04	-.32**	.16	-.00	.26*	-.12	.80**	.76**	.46**	.70**	-		7.35 (3.47)	1.65	2.14
12. HRV groups	-.01	-.17	-.02	-.14	.14	.10	.15	.24*	.01	.11	.05	-	1.51 (.50)	-.03	-2.05

*SD*, Standard deviation; *SE*, Standard error.

*p < .05

** *p* < .01.

### Mixed model analysis of variance

A two-way 2 (HRV group: reduced or increased) x 5 (maltreatment form: physical/emotional/sexual abuse or emotional/physical neglect) mixed ANOVA with repeated measures on the maltreatment form variable was performed. Mauchly’s test of sphericity was significant (*W* = .479, *X*^*2*^ (9) = 55.55, *p* < .001); therefore, the Greenhouse-Geisser correction was used for the within-subjects factor effect (ε = .72).

There was a significant main effect of childhood maltreatment forms experienced by mothers, *F*(2.86, 220.51) = 24.42, *p* < .001. Bonferroni post hoc analyses indicated that mothers experienced significantly more emotional neglect than physical abuse (*p* < .001), emotional abuse (*p* < .05), sexual abuse (*p* < .001) and physical neglect (*p* < .001) during childhood. Emotional abuse was the second most common form of maltreatment among participants (*p* < .001), and physical neglect was the third (*p* < .001). Physical and sexual abuse were mothers’ two least frequent forms of childhood maltreatment (*p* < .001).

**Fig 1:**
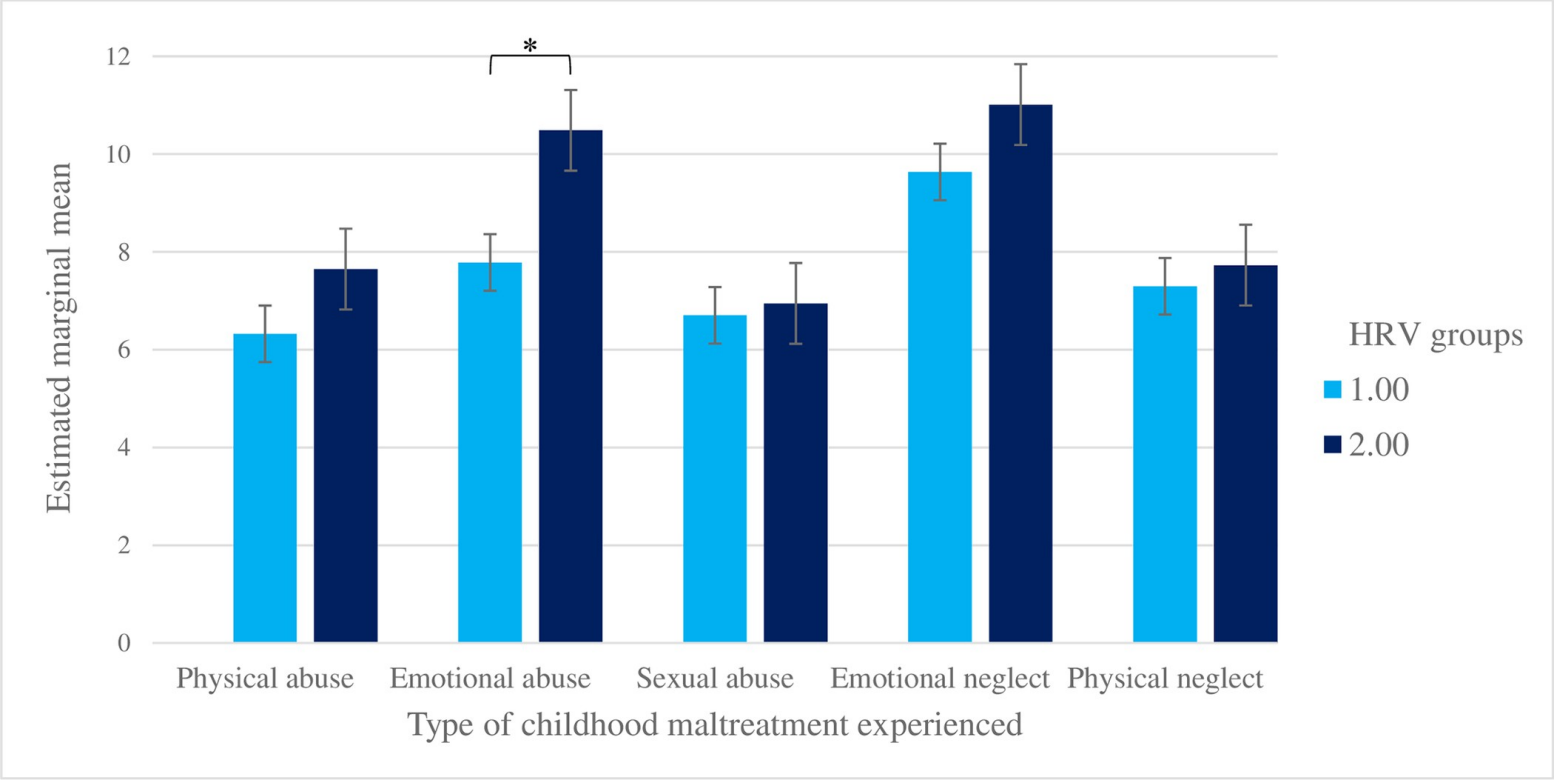
The severity of childhood maltreatment experienced by HRV group. HRV group 1 includes mothers whose HRV decreased while viewing children’s emotional facial expressions compared to viewing landscapes. HRV group 2 includes mothers whose HRV increased. Childhood maltreatment ranged from 5 to 25, with higher scores indicating a more severe trauma. *p < .05.

No significant main effect of HRV was found, indicating that the overall HRV difference between both groups is not significant, *F*(1, 77) = 1.66, *p* = .20. However, a marginal interaction was found between types of childhood maltreatment experienced by mothers (within-group factor) and their HRV change (between-group factor), *F*(2.86, 220.51) = 2.51, *p* = .06. The partial eta squared of .03 indicated a small to medium effect size. As indicated in Table 2 and illustrated in Fig 1, Bonferroni post hoc tests suggested that mothers with increased HRV had experienced more emotional abuse during childhood than mothers with decreased HRV when viewing children’s emotional facial expressions (*p* < .05).

**Table 2:** The severity of childhood maltreatment experienced by mothers according to their HRV group.

Childhood maltreatment forms	HRV groups *N*	1 (reduced HRV) 39	2 (increased HRV) 40	Total 79	*p*
Physical abuse	Mean (*SD*)	6.26 (3.73)	7.50 (4.37)	6.89 (4.09)	.18
Emotional abuse	Mean (*SD*)	7.69 (3.76)	10.23 (6.45)	8.97 (5.41)	.04
Sexual abuse	Mean (*SD*)	6.72 (3.32)	6.80 (4.23)	6.76 (3.79)	.92
Emotional neglect	Mean (*SD*)	9.63 (4.22)	10.76 (5.73)	10.20 (5.04)	.32
Physical neglect	Mean (*SD*)	7.18 (3.07)	7.53 (3.86)	7.35 (3.47)	.66

## Discussion

In the present study, we first examined whether mothers with a history of childhood maltreatment present cardiovascular dysregulation when perceiving facial expressions of emotions in children, using HRV as a measure. Our findings revealed that exposure to images of children displaying different emotions triggered two types of responses in mothers. Some mothers experienced a decreased HRV, indicating a stress-like reaction dominated by the SNS (higher cardiovascular reactivity). Others showed an increase in HRV, suggesting activation of the PNS and a state of relaxation (diminished cardiovascular reactivity). Secondly, we investigated the role of childhood maltreatment in mothers’ cardiovascular responses when exposed to children’s facial expressions displaying different emotions. Results showed a marginal interaction between mothers’ HRV and childhood maltreatment history. More specifically, the severity of emotional abuse experienced during childhood was greater for mothers with increased HRV during the task than for mothers with decreased HRV. Other forms of maltreatment had no influence on mothers’ physiological responses.

It is important to approach the following results with caution, as the observed interaction was marginally significant. However, because the effect size was small to medium, and in light of a rising call in science to go “beyond the *p* < 0.05”, we believe our study brings important knowledge to the field [31, 32]. Our results are another indication that for a group of mothers with a severe history of maltreatment, children’s emotions trigger disengagement. Many meta-analyses have established a relationship between a history of maltreatment and less sensitive behaviors toward the child [22, 33]. The role of emotion recognition in this relationship was highlighted in a study indicating that for mothers with a history of severe maltreatment, good recognition skills were related to a decrease in sensitivity [25]. Research must now explore the mechanisms related to this difficulty in order to break the cycle of maltreatment in which child neglect plays an important role. The physiological reactions to stress stimuli have been under the radar for a while in studies on maltreatment [34]. Still, children’s facial emotions have only recently been considered a stimulus that could induce a stress reaction [35]. Our results align with those of Reijman et al. [20] in that they suggest that mothers’ cardiovascular responses to children’s emotional signals are affected by the severity of their childhood maltreatment experiences. Our results align with those of Reijman et al. [20] in that they suggest that mothers’ cardiovascular responses to children’s emotional signals are affected by the severity of their childhood maltreatment experiences. However, we specifically observed this trend in cases of emotional abuse and not across all forms of maltreatment.

Mothers in our study who were subjected to more severe emotional abuse during childhood displayed signs of sympathetic hypoarousal. These results align with previous research, indicating that higher levels of childhood maltreatment are linked to reduced cardiovascular reactivity to stress tasks [13–16, 36]. Emotional abuse is strongly associated with other forms of maltreatment and may even be a fundamental aspect of all types of abuse and neglect [37]. In our sample, it was strongly correlated to all the other forms of abuse and neglect. This could explain why emotional abuse was the only type of maltreatment that impacted the physiological responses of mothers in our study, mainly as we used a repeated-measure design to control for the shared variance between the five types of maltreatment.

On the other hand, blunted cardiovascular responses have been linked to poorer behavioral regulation, suggesting lower levels of behavioral engagement in mothers during our experiment [38]. A recent study revealed a cluster of adults with lower stress responses across all cardiac parameters, with higher exposure to childhood adversity and increased levels of behavioral disengagement and depression [39]. Emotional abuse encompasses a range of negative experiences, such as spurning, terrorization, isolation, exploitation/corruption, emotional unresponsiveness, and neglect of mental health, medical, or legal needs [40]. These experiences can significantly influence an individual’s self-worth, causing internalized problems as well as disturbing the nervous system [41, 42]. Our findings support the idea that early stressful life events, particularly emotional abuse, can lower the threshold of stress severity needed to trigger responses of disengagement [43].

Other studies using infants’ emotional signals found no impact of childhood maltreatment on parents’ physiological responses [18, 19]. This discrepancy may arise from the usage of different stimuli. As our stimulus targets parental visual perception of emotion and theirs targets parental auditory perception of emotion, they presumably elicit distinctive reactions in participants.

The parent’s level of sensitivity is a crucial factor in the parent-child relationship. Ainsworth [44] described sensitivity as having three components: the parent’s ability to 1) perceive the child’s signals, 2) interpret them correctly, and 3) respond promptly and appropriately. According to our results, mothers are likely to experience cardiovascular changes during the initial stage of Ainsworth’s sensitivity model. This could hinder their ability to meet the following model components. Parents’ physiological responses to their children depend on their background and the context of the signals. Augustine and Leerkes [45] conducted a revealing study on this subject. Their research measured maternal reactions to children using RSA and skin conductance. The study showed that mothers’ physiological reactivity varied depending on their infants’ emotional signals. For infants displaying higher levels of distress, maternal RSA withdrawal following a video of their infants crying in response to a fearful task was related to more sensitive behavior. However, a physiological augmentation during the task was related to greater sensitivity.

To our knowledge, no study has examined parents’ ability to identify children’s emotions based on their cardiovascular reactivity and maltreatment history. However, it has been shown that adults who have experienced childhood maltreatment struggle to recognize negative emotions such as anger, fear, and sadness [46]. It is, therefore, plausible that recognizing children’s emotions might be more demanding for parents with a history of childhood maltreatment. Parents’ cardiovascular reactivity could intensify this difficulty, making it even more challenging to identify their children’s emotions. Accurately identifying a child’s emotional state is crucial to effective parenting [47–49]. Thus, the physiological dysregulation caused by childhood maltreatment may pose a risk factor for maladaptive parenting behaviors [50, 51]. Further research is needed to comprehend this matter thoroughly.

### Strengths and limitations

The current study provides valuable insights into the impact of children’s facial expressions of emotions on the cardiovascular response of parents with a history of childhood maltreatment. Actual knowledge of how parents who have experienced childhood maltreatment respond to their children’s emotions relies on their reactions to vocalized emotions. However, preschool-aged children often express their emotions initially through facial expressions before externalizing them. Hence, our study was centered on the premise that parents could physiologically respond to children’s emotional facial expressions. Additional research is required to further our understanding of the interplay between childhood maltreatment, parental cardiovascular reactivity in response to emotions conveyed by their child, and parenting.

Certain limitations should be considered. We used RMSSD as an indicator of parasympathetic activity, a measure commonly used in the literature [6, 20]. This time value allowed us to make inferences about SNS activity. From a methodological point of view, it would be advisable for future studies to include a direct measure of SNS, such as the pre-ejection period (PEP). Additionally, issues related to attention or visual acuity might have led to a misperception of stimuli during the task, which could have impacted participants’ cardiac data. Respiration and other physiological processes are also potential confounds for HRV [52]. Therefore, measuring and controlling for these variables would have been preferable to minimize study bias. Future studies with a combination of physiological measures could help replicate our data. Also, childhood maltreatment was reported retrospectively, which is influenced by subjective interpretation. A recent systematic review and meta-analysis revealed that retrospective and prospective measures of childhood maltreatment showed poor agreement, implying that they might identify different populations [53]. As a result, these measures cannot be used interchangeably. Since our study focuses on parents’ adaptation to their child, retrospective measures might be more suitable. For instance, self-reported measures of childhood maltreatment are associated with a higher risk of psychopathology, whereas official court records are weakly so if not confirmed by subjective appraisal [54]. In addition, the Childhood Trauma Questionnaire (CTQ) does not collect data on the age of exposure to childhood maltreatment, which is likely to influence physiological responses in adulthood. However, it measures the severity of maltreatment, an important predicting factor for physiological responses to stress [55]. Additionally, the images used in the study depicted only Caucasian children, as diverse images weren’t available when the images were selected from the CAFE database in 2016. However, this limitation is not expected to significantly impact the study’s results since the research did not involve mothers’ ability to recognize children’s emotional facial expressions. Moreover, the study’s correlational nature makes it impossible to draw causal links between variables. Our sample was relatively small and comprised of mothers only, which limits the generalizability of the results. The sample size might have limited the power of our analyses. A G power analysis indicated that we needed at least 84 participants to identify a small effect size. Initially, we had 104 participants, but some had to be excluded due to medication or technical issues, which reduced our sample size to 79 participants. This reduction in sample size may have brought us to the brink of finding a significant effect. We highly encourage fellow researchers to replicate these less significant findings, as they could offer valuable insights into this subtle phenomenon.

To gain a more comprehensive understanding of the impact of childhood maltreatment experiences on parental cardiovascular responses, future research should include fathers and investigate whether they experience similar effects as mothers. It would also be interesting for future projects to examine the cardiovascular responses of parents who have experienced childhood trauma based on the emotional valence (neutral, positive or negative) of children’s facial expressions.

## Conclusion

The findings of this study raise the possibility that mothers’ cardiovascular responses to children’s emotional cues may be influenced by their childhood maltreatment experiences. Mothers who had experienced more severe emotional abuse during childhood seemed more likely to show an increase in HRV when watching children’s emotional facial expressions, which could be associated with lower levels of behavioral engagement. This hyperreactivity of the nervous system in mothers could interfere with their ability to accurately recognize their children’s emotions and thus impact their level of sensitivity when interacting with their children. Our findings highlight the significance of analyzing distinct types of maltreatment individually, despite their tendency to occur together, as each individual may experience them differently. Moreover, our research underscores the importance of further investigation into the connection between parents’ cardiovascular reactions, their capacity to perceive their children’s emotions accurately, and their ability to adjust their parenting behavior accordingly. Perhaps the key to breaking the intergenerational maltreatment cycle lies in parents’ physiological responses to their child’s emotional cues. By recognizing and addressing their own responses, parents can create a safe and nurturing environment for their children, preventing the transmission of difficult parenting from generation to generation.

## Supporting information

S1 File (SAV)
